# On a Gland, Hitherto Not Accurately Described, in the Interior of the Tip of the Tongue

**Published:** 1846-01

**Authors:** 


ART. IX.—*Ueber eine bis jetzt noch nicht näher beschriebene Drüse im innern der Zungenspitze.* Von A. NÜHN, Doct. Med. &c.—*Mannheim*, 1845.

*On a Gland, hitherto not accurately described, in the interior of the tip of the Tongue.* By A. NÜHN, M.D., &c.—*Mannheim*, 1845. 4to, pp. 8.

MANY less modest authors would have made a larger book than this, to proclaim the discovery of an organ, visible to the naked eye, in the human body. We have found Dr. Nühn's description of these glands exactly true to nature; and they are so easily dissected, that all who are interested in anatomy will, with a few helps, find them for themselves. When the mucous membrane is removed from a slightly elevated elongated oval space which is always seen about two lines from the median line, on the under surface of the apex of the tongue, a layer of longitudinal muscular fibres is at once exposed. If, next, these, which form part of the inferior lingualis and of the anterior fibres of the styloglossus, be reflected or removed, the gland is seen just under the canine artery, outside the expanding branches of the lingual (5th) nerve, just anterior to the edge of the hypoglossus. The gland measures from seven to ten lines in length, from three to four and a half in breadth, and from one and a half to two and a half in thickness. In structure and general aspect it agrees with the larger labial, lingual, and other similar glands. It has many ducts which open through the mucous membrane over it, by irregularly placed orifices, each of which is surrounded by a slightly elevated ring. The author supposes it to be a mucous gland; and that it may be in some way subservient to speech, for he finds it in no animal, except man, and the orang, though he has searched for it in very many species. In the orang it is rather larger than in man.

